# Complete Genome Sequence of *Delftia tsuruhatensis* CM13 Isolated from Murine Proximal Colonic Tissue

**DOI:** 10.1128/genomeA.01398-16

**Published:** 2016-12-15

**Authors:** Azadeh Saffarian, Céline Mulet, Régis Tournebize, Tomoaki Naito, Philippe J. Sansonetti, Thierry Pédron

**Affiliations:** a Unité de Pathogénie Microbienne Moléculaire, INSERM U1202, Institut Pasteur, Paris, France; b Unité de Pathogénie Microbienne Moléculaire, INSERM U1202, Imagopole Citech, Institut Pasteur, Paris, France; c Chaire de Microbiologie et Maladies Infectieuses, Collège de France, Paris, France

## Abstract

We report here the complete genome sequence of *Delftia tsuruhatensis* CM13, isolated from murine proximal colonic tissue. The genome assembly using PacBio single-molecule real-time sequencing resulted in a single scaffold of 7.19 Mb.

## GENOME ANNOUNCEMENT

We recently identified aerobic, nonfermentative bacteria located inside murine intestinal colonic crypts (1). They belong mainly to the genus *Acinetobacter*, and *Delftia* and *Stenotrophomonas* spp. were also identified. The Illumina HiSeq 2000 technology (paired-end libraries) was previously used to sequence two strains of *Acinetobacter* (*A. parvus* and *A. radioresistens*) and one strain of *Stenotrophomonas maltophilia* isolated from proximal colonic tissues from C57BL/6 mice (2). Here, we utilized PacBio single-molecule real-time (SMRT) sequencing technology (3) to generate a *de novo* assembly of the complete genome sequence of *Delftia tsuruhatensis* CM13. 

Bacteria were isolated as already described (2), and genomic DNA was extracted by the Wizard genomic DNA purification kit, according to the manufacturer’s instructions (Promega), followed by three extractions with phenol-chloroform. A single library was prepared for *D. tsuruhatensis* CM13 and run on two SMRT cells on the PacBio RS II platform (https://www.gatc-biotech.com). SMRT sequencing of the *D. tsuruhatensis* CM13 strain resulted in 52,431 reads with a mean read length of 13,539 bp, totaling 709,903,604 nucleotides with an average coverage of 90.21×. The initial assembly was conducted using the Hierarchical Genome Assembly Process (4). The final complete genome resulted in a single contig of 7,195,716 bp, with a total G+C content of 66.3%.

Automated annotation was performed using the RAST server (5) (http://rast.nmpdr.org). RAST predicted 6,669 coding sequences (CDSs), of which 97 encode RNA regions. Specifically, of the 97 RNA regions, 15 encode rRNA and 82 encode tRNA. The coding sequences were classified into 512 subsystems, with the most abundant systems being those involved in the metabolism of amino acid derivatives (*n* = 594 CDSs) and carbohydrates (*n* = 512); membrane transport (*n* = 437); cofactors, vitamins, prosthetic groups, and pigments (*n* = 386); protein metabolism (*n* = 314); fatty acids, lipids, and isoprenoids (*n* = 297); and virulence, disease, and defense (*n* = 219).

### Accession number(s).

The genome sequence of *D.tsuruhatensis* CM13 was deposited at DDBJ/EMBL/GenBank under the accession number CP017420.
